# Long-term outcomes of the modified Nirschl technique for lateral Epicondylitis: a retrospective study

**DOI:** 10.1186/s12891-021-04079-x

**Published:** 2021-02-19

**Authors:** Soonchul Lee, In-Tae Hong, Soohyun Lee, Tae-sup Kim, Kyunghun Jung, Soo-Hong Han

**Affiliations:** 1 Department of Orthopaedic Surgery, CHA Bundang Medical Center, CHA University School of Medicine, Seongnam-si, Gyeonggi-do 13496 Republic of Korea; 2 Department of Orthopaedic Surgery, Dongtan Sacred Heart Hospital, Hallym University, Hwaseong-si, Gyeonggi-do 18450 Republic of Korea

**Keywords:** Lateral epicondylitis, Surgery, Wrist extension, Strength, Clinical outcome

## Abstract

**Background:**

Although the Nirschl technique was introduced approximately 40 years ago, only limited information is available about the long-term results, especially concerning extensor power changes after surgery.

The purpose of this study was to investigate long-term clinical results of surgical treatment of lateral epicondylitis using the modified Nirschl technique. The main outcome variable was muscle strength for wrist extension because the extensor origin was not reattached after removal of the degenerative extensor tendon.

**Methods:**

Data from 99 patients who underwent surgical lateral epicondylitis treatment between 2007 to 2012 were included in the study. The mean follow-up period was 8.5 years (5 to 10, ± 1.1 years) and the mean age at surgery was 44.8 years (32 to 70, ± 9.8 years). The surgeries were performed using the modified Nirschl method and did not include extensor origin reattachment. Outcome measurements included the Visual Analogue Scale (VAS) score, Disabilities of the Arm, Shoulder and Hand (DASH) score, the MAYO elbow performance score, and Nirschl and Pettrone’s grades. Wrist extension and grip strength were analyzed using a digital handgrip dynamometer (microFET2TM system) and JAMA hand dynamometer.

**Results:**

Mean time required to return to work was 2.4 months after surgery. At the last follow-up after surgery, the mean VAS score had significantly improved, from 4.9 to 1.1. Mean MAYO elbow performance scores significantly improved, from 64 to 90, and mean DASH scores improved from 50 to 13. The Nirschl and Pettrone’s grades were 80% rated as ‘excellent’ and 16% rated as ‘good’. After adjusting for power differences between the dominant and non-dominant arms, the difference between wrist extensor power of the operated elbow and the non-operated opposite elbow at the final follow-up was not statistically significant. No patients complained about wrist extension weakness.

**Conclusion:**

Although reattachment of the extensor origin was not performed during the modified Nirschl surgical technique, there was no significant weakness in wrist extension power and the long-term follow-up revealed favorable clinical results.

**Level of evidence:**

Level IV (case series). Retrospective study.

**Supplementary Information:**

The online version contains supplementary material available at 10.1186/s12891-021-04079-x.

## Background

Lateral epicondylitis is caused by degeneration of part of the extensor tendon origin [1]. This functionally limiting and painful condition is relatively common among working-age individuals in the population. Studies have found the prevalence of lateral epicondylitis to be 1–4%; the prevalence is highest in subjects 45–64 years of age [2, 3].

Non-operative therapy is the mainstay for lateral epicondylitis treatment. Many patients recover with non-operative treatment (e.g., activity modification with a brace, anti-inflammatory medications, local corticosteroid injections, physiotherapy, and extracorporeal shock wave therapy) [4–6]. Surgical treatment is indicated for those who do not respond to the use of conservative measures, and studies have found that the proportion of surgically treated patients is 2–8% [7–9]. In developed countries, the proportions of patients > 65 years of age who receive surgical treatment for lateral epicondylitis has significantly increased as life expectancies have increased [8].

Different surgical techniques for lateral epicondylitis treatment have been described, such as debriding the pathologic extensor origin with or without repair of the extensor tendon, releasing the posterior interosseous nerve, denervation of the lateral epicondyle, and anconeus rotation [10–15]. In 1979, Nischl described a surgical technique in which the interface between the extensor carpi radialis longus and extensor aponeurosis is incised, the abnormal granulation tissue associated with the extensor carpi radialis brevis is released and resected, and decortication and drilling multiple holes at the lateral epicondyle is performed without reattachment of the extensor origin [13]. However, although this technique was introduced approximately 40 years ago, only limited information is available about the long-term results of the Nirschl technique, especially with regard to changes in extensor power after surgery [16–18]. Most previous studies have evaluated functional outcomes using subjective approaches such as functional scoring systems, wrist and elbow range of motion (ROM) measurement, and measurement of grip strength of the affected limb. However, how the extensor power of the wrist is affected as a long-term result of using the Nirschl technique without extensor origin reattachment remains unanswered.

By our clinical experience, surgically treated patients mostly show a definite loss in function, especially in wrist and finger extension power, caused by intolerable pain preoperatively. Therefore, goal of surgery was focused more on thorough removal of the pain source and making the good nutritional bed for spontaneous regeneration of the defect site and less on reattachment of the remnant extensor tendon to the origin. Even though reattachment was not made, most patients showed definite improvement of function clinically. Moreover, it is technically demanding to reattach the remnant extensor tendon to the origin and too much tension on the reattachment site might cause pain, limitation of wrist flexion or failure of the suture.

Consequently, our hypothesis was that even though the extensor origin is not repaired, there would be no long-term negative impact of wrist extension strength after surgery. To examine this hypothesis, we analyzed the long-term results of the modified Nirschl procedure by measuring extensor power using a dynamometer.

## Methods

### Patients and demographics

The Institutional Review Board of our hospital approved the study protocol. A retrospective review of medical records was performed for a total 144 patients who underwent surgical lateral epicondylitis treatment during 2007–2012.

Diagnosis of lateral epicondylitis was based on history and physical findings, including pain and local tenderness around the lateral epicondyle. Patients reported symptoms including diminished wrist extension and hand grip power due to pain and painful resistance against extension of the wrist or third finger. Joint and bony lesions (e.g., osteophyte or osteoarthritis) were identified using plain radiography and ultrasonography. Radial tunnel syndrome and radiohumeral plica syndrome were ruled out based on the results of palpation of the radial tunnel area and radiocapitellar joint.

**Fig. 1:**
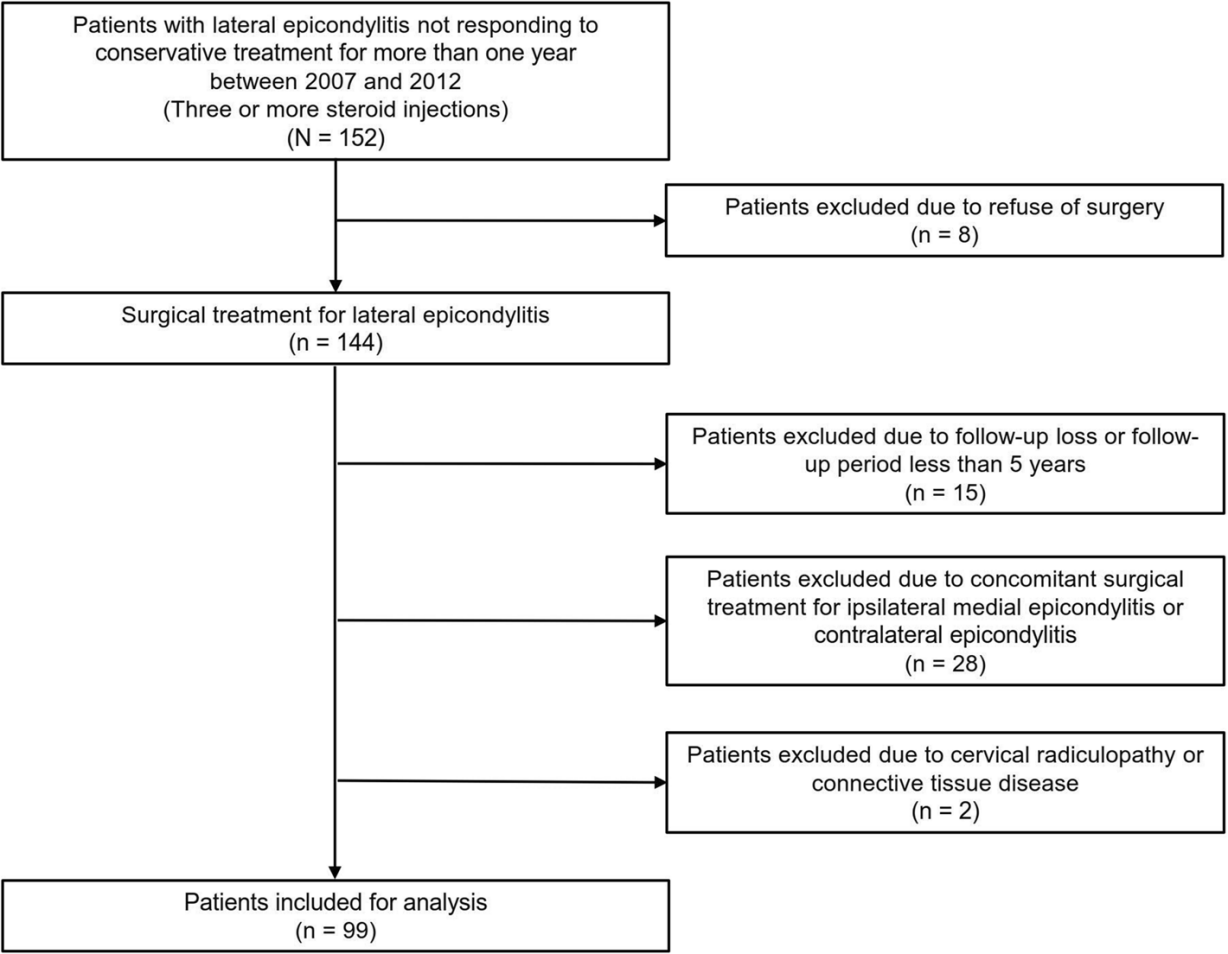
Flowchart of patient selection

The indication for lateral epicondylitis surgery was no response for at least 1 year to non-operative treatment, including anti-inflammatory medication and two or more steroid injections. Data from 15 patients with a follow-up time < 5 years were excluded from the analysis. Twenty-eight patients were excluded who had simultaneously undergone surgical treatment for ipsilateral medial epicondylitis or contralateral lateral or medial epicondylitis. Data from an additional two patients who had cervical radiculopathy and connective tissue disease diagnosed before and after surgical treatment were also excluded. Finally, 99 patients who had no associated upper-limb disease and were affected by lateral epicondylitis in only one arm were included in this study (Fig. 1).

There were 39 males and 60 females in the study population. The mean age at surgery was 44.8 years (32 to 70, ± 9.8 years). The mean duration of symptoms before surgery was 22 months (13 to 120, ± 10.2 months), and the corticosteroid was injected 3.1 times (2 to 10, ± 0.6 times) before surgery. The disease affected the dominant arm of 64 patients and the non-dominant arm of 35 patients. Fifty-two of 99 patients were manual laborers, which required repetitive wrist flexion and pronation. This group included those who worked in construction and manufacturing and as mechanics and bus drivers. Thirty-two patients were homemakers. Fifteen patients were clerical workers. Twenty-five of the 99 patients engaged in regular sports activities. Seven played golf, 5 played tennis, 4 were swimmers, 8 exercised at a fitness center, and 1 engaged in archery.

### Operative technique

**Fig. 2:**
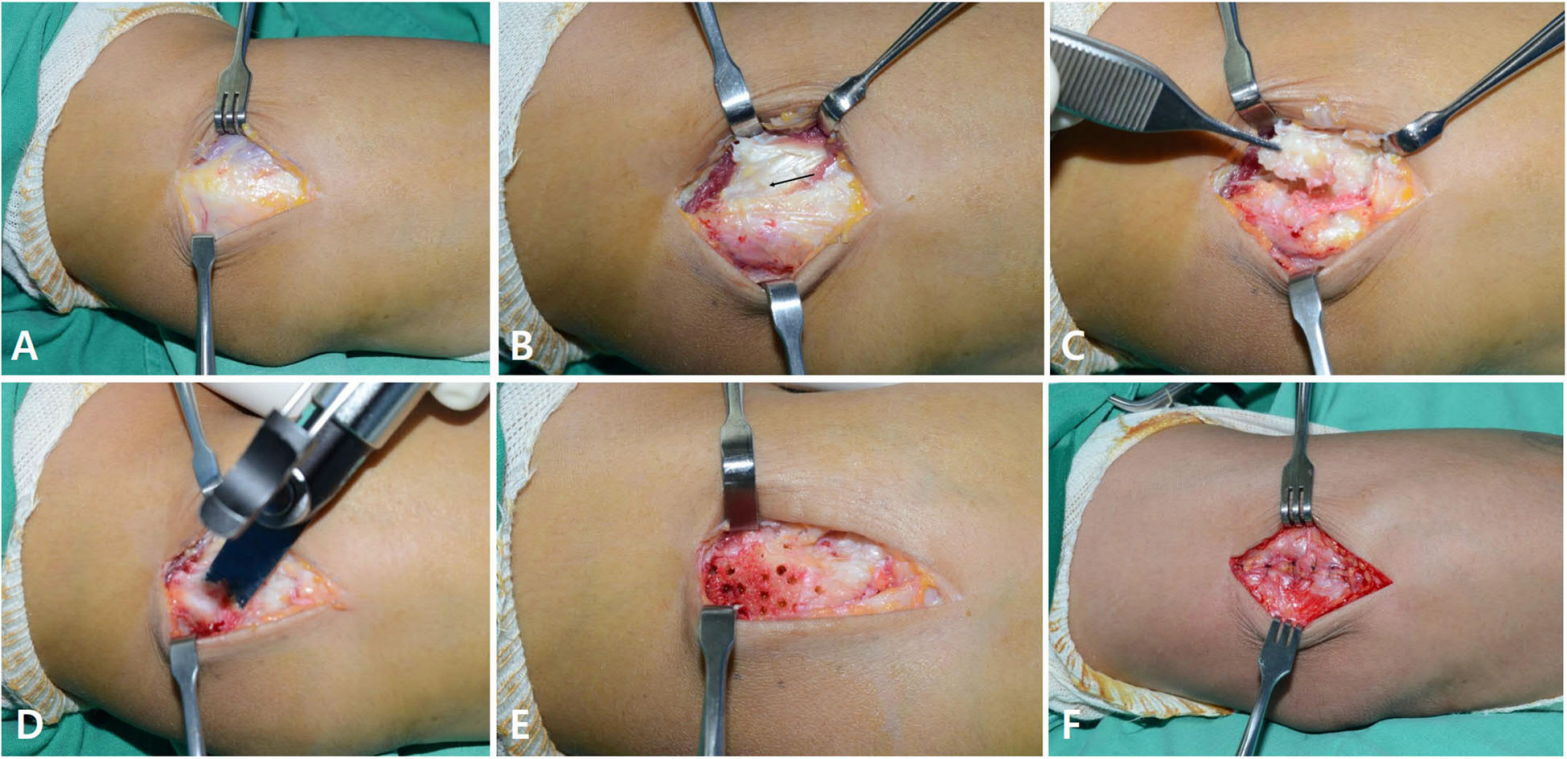
Surgical procedure for lateral epicondylitis. **a**. As subcutaneous tissues are elevated, the fascia of the extensor muscle mass is exposed. The extensor aponeurosis and muscle fibers of extensor carpi radialis longus (ECRL) and extensor carpi radialis brevis (ECRB) are visible. **b**. The proximal part of the interval between the ECRL and extensor aponeurosis is split and the ECRL is retracted anteriorly. Pathologic tendinopathy tissue of the ECRB (black arrow) is visually identified by its characteristic dull grayish color; it is usually edematous and friable. **c**. Excision of the pathologic and abnormal appearing tissue involving the ECRB, EDC tendon, and richly innervated periosteum at the site of the muscle’s origin is performed elliptically, leaving the normal tissue of the attachment to the lateral epicondyle. **d**. An oscillating saw is used to decorticate the lateral condyle. **e**. Drilling multiple small holes on the lateral condyle is performed to create a sufficient vascular bed. **f**. Anatomic repair of the interval between the posterior edge of ECRL and the common extensor aponeurosis performed without reattachment of the ECRB to its origin

The same surgeon performed all procedures. General anesthesia and tourniquet control were used for all patients. Each patient was in the supine position with the affected limb on an arm table. The arm was positioned with the shoulder in abduction and internal rotation, so that the anterolateral side of the lateral epicondyle faced the surgeon. A slightly curved longitudinal 5 cm incision was made along the lateral aspect of the distal portion of the humerus, slightly anteromedial to the lateral epicondyle. The fascia of the extensor muscle mass was exposed as subcutaneous tissues were dissected. The extensor carpi radialis longus (ECRL) muscle and extensor aponeurosis were split and retracted to expose the pathological tissue at the origin of the extensor carpi radialis brevis (ECRB) tendons. Pathologic tendinopathy tissues associated with the ECRB tendons were visually identified by the characteristic dull grayish color; they were also usually edematous and friable. Thorough excision of the pathologic and abnormal appearing tissues involving the ECRB and extensor digitorum communis tendons and richly innervated periosteum at the site of the muscle’s origin was performed elliptically. Normal tissue was left attached to the lateral epicondyle. The radiohumeral joint was not exposed. The bony surface exposed after resection of the unhealthy tendon was decorticated using an oscillating saw. Multiple small holes were drilled with 1.2 mm K-wire to create a sufficient vascular bed. This step varied slightly from the original Nirschl technique in that the decortication area was much larger and decortication and multiple drilling were performed in all cases. The surgeon did not try to reattach the extensor origin. Anatomic repair of the interval between the posterior edge of the ECRL and common extensor aponeurosis was performed to allow early post-operative ROM. The tourniquet was deflated, and hemostasis was performed before closure because post-operative hematoma can be a significant negative outcome. After placing ordinary subcutaneous sutures, a running subcuticular skin suture with absorbable suture (4–0 coated undyed Vicryl) was done (Fig. 2).

### Post-operative care

During the post-operative period, the arm of each patient was immobilized using a long-arm splint in elbow extension. In patients with tolerable pain, the splints were removed (minimally 3 days after surgery). After splint removal, each patient was allowed to actively use the elbow (e.g., for computer use, writing, and activities of daily living). However, all patients were instructed to avoid using the extremity for lifting or carrying. No other specified physical therapy was performed after surgery. Return to sports activities (e.g., tennis and golf) was permitted at 12 weeks post-operatively if the full ROM and strength was achieved. The recommended follow-up times were at post-operative 1 month, 3 months, 6 months, and annually. However, most patients did not comply with the recommendations to visit after symptoms improved. The patients were contacted by phone to revisit the hospital for evaluation.

### Evaluation

#### Clinical scoring

ROM of the elbow, grip strength, wrist extension strength, the pain visual analog scale (VAS) score (0–10 points), MAYO elbow performance score (0–100 points) [19], Disabilities of the Arm, Shoulder and Hand (DASH) score (0–100 points) [20], the Nirschl and Pettrone’s grade (rated based on four categories: excellent, good, fair, and poor), and duration required to resume daily activities and work for each patient were evaluated pre-operatively and at the final follow-up visit.

#### Strength measurement

Extension strength of both wrists were measured using a digital handgrip dynamometer (microFET2TM System, Hoggan Health Industries Inc., Salt Lake City, UT, USA) and a customized method. A special cradle was designed and manufactured to hold the dynamometer firmly in place. The cradle was installed on a closed system specially designed and manufactured to accurately evaluate wrist extension power.

**Fig. 3:**
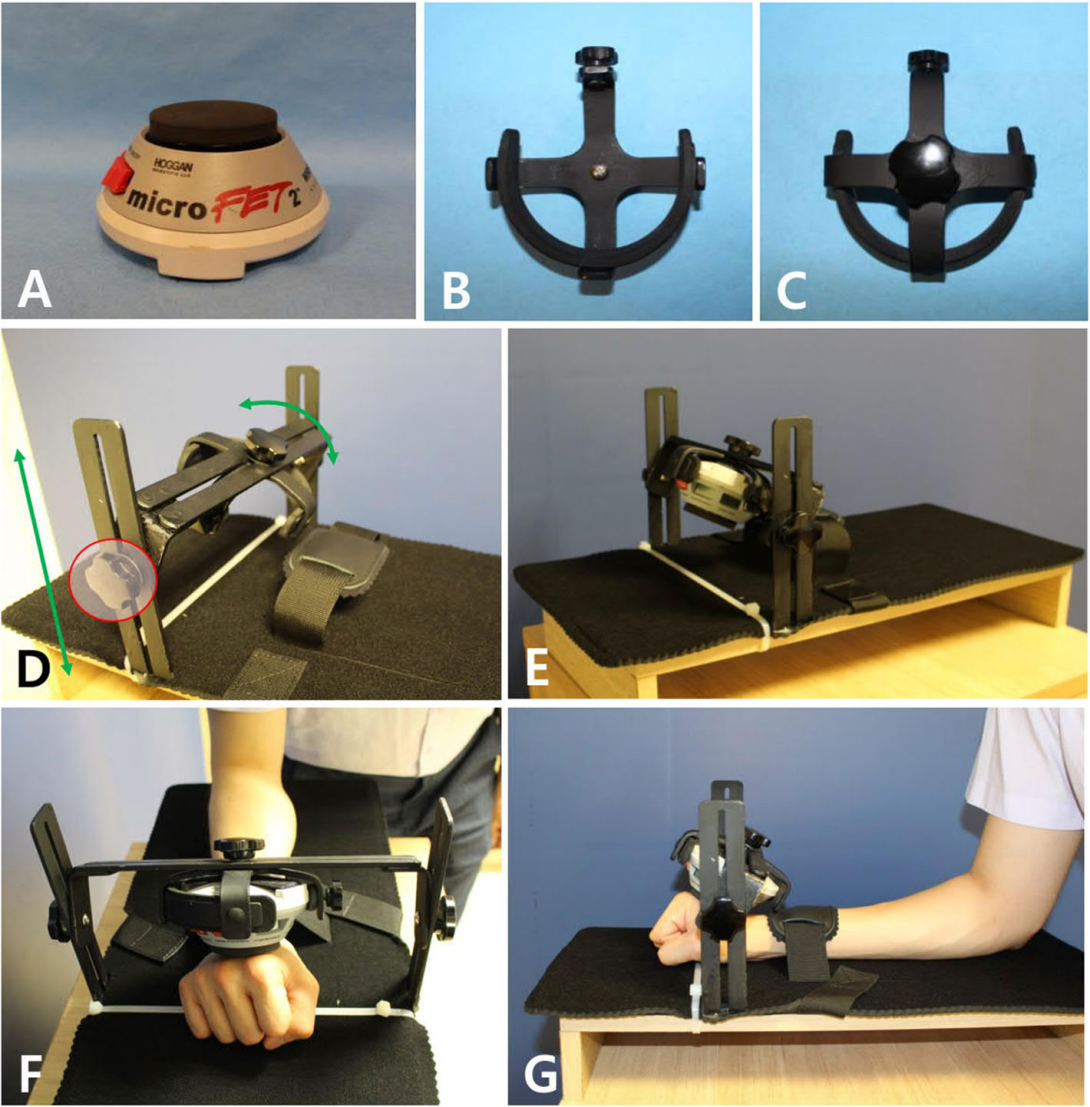
Evaluation of wrist extensor strength. **a**. Digital hand-held dynamometer (microFET2TM System, Hoggan Health Industries Inc., Salt Lake City, UT, USA). **b** & **c**. Top and bottom views of custom-designed holder. Semi-circle shaped liner consists of rubber to tightly hold the dynamometer. The screw on the one end tightens the dynamometer into the liner. **d**. Screws at both sides (red circle) of the cradle function to lever and to tilt the holder (green arrows). **e**. Overall view of the custom-designed system that measures wrist extensor strength. **f**. The head of the dynamometer placed perpendicular to the long axis of the forearm over the long finger metacarpal at the level of the metacarpal neck. The wrist is stabilized onto the plate. The subject is asked to extend the wrist with maximal force for 5 s, and the maximal value is recorded as the muscle power. G. The subject’s forearm laid flat on the plate with the wrist in pronation and neutral flexion-extension position and the shoulder in slight abduction and neutral rotation. Wrist is stabilized firmly onto the plate using the wrist strap

Each patient’s forearm was laid flat on the board with the wrist in pronation and neutral flexion-extension position, and the shoulder in slight abduction and neutral rotation. The head of the dynamometer was placed perpendicular to the long axis of the forearm over the third metacarpal at the level of the metacarpal neck area. The wrist was stabilized using a band that was attached to the board. The patient was asked to extend the wrist with maximal force for 5 s, and the maximal value was recorded as muscle power (Fig. 3, Supplemental Video 1).

**Fig. 4:**
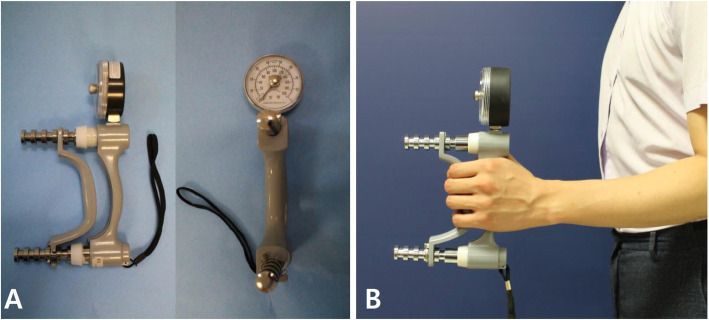
Evaluation of grip strength. **a**. The Jamar hand dynamometer (Hydraulic Hand Dynamometer®, 5030 J1, Sammons Preston, Bolingbrook, IL, USA). **b**. With the forearm in neutral position and the shoulder in adduction and neutral rotation, the subject is asked to squeeze the handle of the dynamometer with maximal force for 5 s and the maximal value is recorded as the muscle power

Grip strength was evaluated using a Jamar hand dynamometer (Hydraulic Hand Dynamometer, 5030 J1, Sammons Preston, Bolingbrook, IL, USA). With the forearm in neutral position and the shoulder in adduction and neutral rotation, the patient was asked to squeeze the handle of the dynamometer with maximal force for 5 s. The maximal value was recorded as muscle power (Fig. 4, Supplemental Video 2).

All strength measurements were performed in triplicate. Two senior orthopedic residents (third or fourth grade) and one fellowship trainee of elbow and hand surgery division performed the strength evaluations. The mean values were used for the final analyses.

Based on a literature review, we assumed that wrist extensor power was 10 and 11% greater for the dominant arm compared to the non-dominant arm in males and in females, respectively [21]. We also assumed that grip strength was 6 and 8% greater for the dominant arm compared to the non-dominant arm in males and in females, respectively [22]. Consequently, when comparing both arms, the dominant arm muscle power values for wrist extension and grip strength were adjusted accordingly to compare with the non-dominant arm, and vice versa.

### Statistical analysis

Mean and standard deviation values were calculated from the numerical data. Kolmogorov-Smirnov tests were used to determine whether the data for continuous variables met the assumption of a normal distribution. Data sets were compared using paired t-tests. Clinical scores were compared between the pre-operative state and final state at last follow-up. ROM, extensor power and grip strength of the operated arm were also compared to the non-operated arm at final follow-up. The Kappa values were calculated for interobserver reliability of the strength measurements. The statistical software SPSS for Windows Version 18.0 (SPSS, Chicago, IL, USA) was used for all statistical analyses. Statistical significance was determined at the *p* < 0.05 level.

## Results

**Fig. 5:**
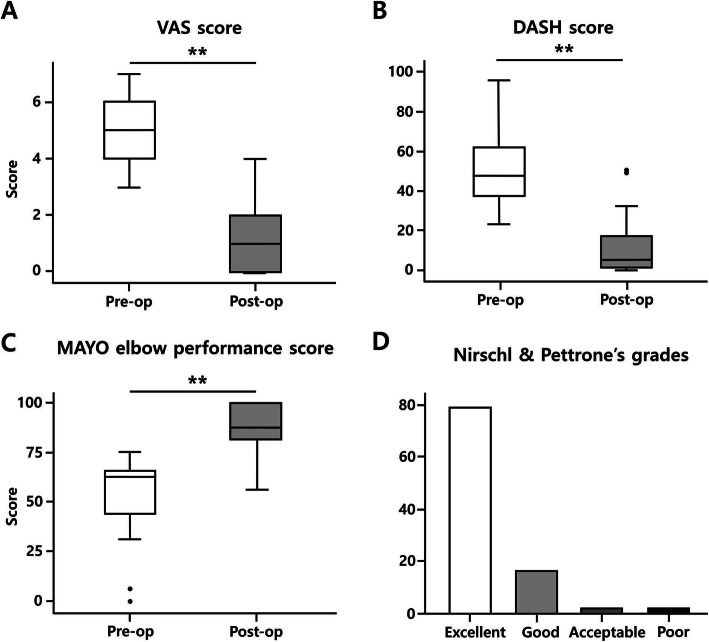
Results for functional scores. **a**. The mean VAS scores showed significant improvement at the last follow-up, from 4.9 to 1.1 (*p* < 0.001). **b**. DASH scores improved from 50 to 13 (*p* < 0.001). **c**. MAYO elbow performance scores improved significantly, from 64 to 90 (*p* < 0.001). **d**. Nirschl and Pettrone’s grades were rated as ‘excellent’ in 79.8% (79 elbows) and as ‘good’ in 16.2% (16 elbows). ** indicates *p* < 0.01

The mean follow-up period was 8.5 years (5 to 10, ± 1.1 years). There was significant improvement in the mean VAS score at the last follow-up visit (from 4.9 to 1.1; *p* < 0.001). The improvement in mean MAYO elbow performance scores was also significant (64 to 90; *p* < 0.001), and mean DASH scores improved significantly, from 50 to 13 (*p* < 0.001). The Nirschl and Pettrone’s grades were 80% (79 elbows) rated ‘excellent’, and 16% (16 elbows) ranked ‘good’. Therefore, the overall success rate of the procedure was 96% (95 of 99 elbows) (Fig. 5). The mean total active ROM was 139 degrees (125 to 145, ± 5.3 degrees) and was not significantly different with the non-operated arm.

**Fig. 6:**
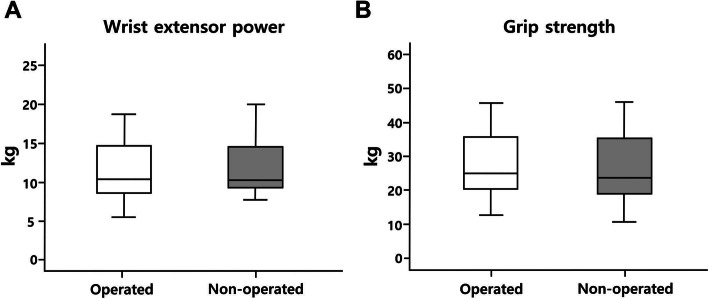
Comparison of operated and opposite wrist extensor strength and grip strengths after adjustment. **a**. No statistical statistically significant difference between the wrist extensor powers of operated versus non-operated elbows (*p* = 0.215). **b**. No statistically significant differences between grip strengths of operated versus non-operated elbow (*p* = 0.155). (Operated: extension power of operated elbow; non-operated: extension power of normal contralateral elbow)

At the final follow-up, the mean wrist extension and grip strengths of the affected side were 11.8 kg and 30.5 kg, respectively. After adjusting extensor power values for differences between dominant and non-dominant arms, the differences between wrist extensor power and grip strength of the operated and the non-operated arms were not statistically significant (*p* = 0.215, *p* = 0.155, respectively) (Fig. 6). There was very good interobserver reliability in the strength measurements. The kappa values of wrist extension and grip strengths were 0.87 and 0.92 in each.

All surgical wounds healed without infection or inflammation. No patient had other complications that required additional surgery (e.g., neurologic symptoms, reflex sympathetic dystrophy, elbow instability, or elbow joint synovial fistula). One patient’s symptoms of lateral epicondylitis recurred, but conservative treatment was administered (i.e., medication) because he refused another surgery. The mean time required to return to work was 2.4 months (1.5 to 6, ± 0.6 months). Two patients stopped work as manual laborers and three patients experienced exercise discomfort because of pain and weakness. No other surgery-related complications were observed.

## Discussion

In this retrospective study, we analyzed data from 99 patients who underwent surgery for lateral epicondylitis after it did not respond to non-surgical treatment. The operative technique was based on the Nirschl method. During the procedure, we did not violate the annular ligament and the detached extensor tendon was not reattached to its origin. After resection of the pathologic tendon, decortication and multiple drilling of the lateral epicondyle were performed to enhance the vascular supply. The clinical results at a minimum 5 years of follow-up revealed statistically significant improved functional scores and muscle strength. At the final follow-up, wrist extensor power was not significantly different from the normal contralateral arm when values were adjusted according to the relative powers of the dominant and non-dominant arms in normal healthy populations [21].

Although there are various techniques for lateral epicondylitis treatment, the main objectives of operative treatment are generally similar. The objectives are to resect pathological tissues, stimulate neovascularization by producing focused local bleeding, and create a healthy scar while minimizing structural damage to surrounding tissues. However, whether it is necessary to reattach the detached extensor tendon to the lateral epicondyle after debridement of degenerative tissue had been not determined.

Early studies in the late 1900s and early 2000s showed concerns about strength deficits after open surgical treatment to lateral epicondylitis. Almquist et al. [10] found 70 to 80% recovery in operated side grip strength than the non-operated side when anconeus transfer was performed. Khashaba et al. [23] used standard Nirschl techniques without drilling or decortication. They reported that at 6 months, the mean extension power was less than that of most unaffected elbows in most patients. Jobe et al. [24] evaluated 39 patients treated using debridement and side-to-side repair of the ECRB. After the recovery period, 36% had limitations with heavy lifting, 50% had grip-dynamometer deficits, and 100% had some degree of isokinetic deficit.

Thereafter, there were studies that presented good results after reattachment of extensor origin. Rosenberg et al. found that 19 of 22 patients with reattachment of the common extensor origin after ECRB release for lateral epicondylitis had satisfactory clinical results [25]. Steven J et al. reported the results for an at least 2-year follow-up period after reattachment of the ECRB to the lateral epicondyle using a suture anchor, after excision of abnormal ECRB fibers [26]. They found that grip strength and pinch strength were 110 and 106%, respectively, compared with the non-operated arm.

Despite these concerns, more recent studies that showed the long-term outcome of the Nirschl technique showed good results. Coleman et al. used the modified Nirschl technique without ECRB reattachment to repair 137 lateral epicondylitis elbows. They found > 90% good to excellent results during long-term follow-up and no significant differences in grip strength between both limbs in any position [16]. Schipper et al. and Dunn et al. also reported highly successful long-term results using the Nirschl technique to treat lateral epicondylitis. However, objective measurement of muscle strength was not evaluated [17, 18]. Our results also indicated that even though there was no reattachment to the common extensor origin, wrist extension power was not reduced compared with the non-operated side.

For an accurate and objective analysis of wrist extension power, we refer to Suzuki et al.’s method [27]. They collected objective measurements of muscle power using microFET2TM and a Jamar hand dynamometer [28, 29]. However, in a preliminary study, we found that measuring wrist extensor power using a hand-held method resulted in only moderate to good inter- and intra-observer reliability (0.54 and 0.76, respectively, data not shown). Therefore, we designed a closed system that can firmly resist wrist extension power, and we validated the reliability of the system using 25 healthy individuals. Inter- and intra-observer reliabilities for the custom-designed system were 0.85 and 0.87, respectively. This result indicated very good reliability. Also, based on previously published data from normal healthy populations, the wrist extension and grip strength muscle powers of the dominant and non-dominant arms were adjusted as described to compare with the opposite arm.

Some previous studies have found decreased extensor power after surgical treatment and recommended reattachment of the extensor origin. However, our results were satisfactory even though extensor origins were not reattached. Some strengths of our study were that only patients who had follow-up periods > 5 years were included and that we evaluated extensor power using an objective dynamometer-based method.

A possible explanation for why there was no long-term weakness in wrist extension strength is as follows. First of all, the previous cadaveric study showed that the body of the ECRB converges with muscular fibers of the EDC at a mean of 68 mm distal to the radio-capitellar joint [30]. Therefore, muscle length can be maintained after resection of the proximal origin of ECRB. Because muscle power was measured at the final follow-up of a mean of 8.5 years, it is possible that fibrous healing had already progressed and filled the gap. Secondly, removal of the common extensor origin and lateral epicondyle during the surgical procedure may not have had significant effects on muscle strength due to the small proportion of footprint area relative to the total origin area.

The strengths of this study included that all patients were treated using the same operative technique. The number of patients was relatively large compared with previous studies. However, it was a retrospective study, and no other surgical techniques were compared to validate that this technique was more effective. Further studies using prospective designs that compare different surgical methods are needed to determine the optimal surgical method to treat patients with lateral epicondylitis.

## Conclusions

This study was the first to use analysis of objective measures to assess wrist extension strength after surgical treatment for lateral epicondylitis. Although reattachment of the extensor origin was not performed, the modified Nirschl surgical technique for lateral epicondylitis showed satisfactory results during the > 5-year follow-up period, without compromise of wrist extensor power.

## Supplementary Information

**Additional file 1: Supplemental Video 1.** Evaluation of wrist extension strength

**Additional file 2: Supplemental Video 2.** Evaluation of grip strength

## Data Availability

The datasets used and/or analysed during the current study are available from the corresponding author on reasonable request.

## Abbreviations

VAS: Visual Analogue Scale
DASH: Disabilities of the Arm Shoulder and Hand
ROM: Range of motion
ECRL: Extensor carpi radialis longus
ECRB: Extensor carpi radialis brevis
